# Care manager organisation in Swedish primary care centres: impact of sick leave and sick leave duration in patients with common mental disorders. A register-based study

**DOI:** 10.1080/02813432.2025.2477150

**Published:** 2025-03-14

**Authors:** Christine Sandheimer, Cecilia Björkelund, Dominique Hange, Christina Möller, Eva-Lisa Petersson, Irene Svenningsson, Gunnel Hensing

**Affiliations:** a Primary Healthcare, School of Public Health and Community Medicine, Institute of Medicine, The Sahlgrenska Academy, University of Gothenburg, Gothenburg, Sweden; b Research, Education, Development & Innovation, Primary Healthcare, Region Västra Götaland, Sweden; c Social Medicine, School of Public Health and Community Medicine, Institute of Medicine, The Sahlgrenska Academy, University of Gothenburg, Gothenburg, Sweden

**Keywords:** Primary health care, sickness absence, sick-listing, collaborative care, care manager, mental disorders, depression, anxiety, stress

## Abstract

**Motivation:**

Primary care centres are the first line of mental health service in Sweden responsible for individuals with mild to moderate severe symptoms of common mental disorders (CMD). The aim was to evaluate impact of sick leave and sick leave duration in patients diagnosed with CMD in primary care centres with a care manager organisation during the first and second year after implementation compared to usual care.

**Methods:**

Register data on sick leave (mean number of net and gross sick leave days) among patients with CMD was obtained per primary care centre from the national social insurance database MiDAS. Two measures of sick leave were used: *impact of sick leave* in total patient population with CMD, and *sick leave duration* among sick listed patients with CMD. Linear mixed-effects regression analysis was performed for cross-sectional differences and longitudinal changes between and within the two groups of primary care centres.

**Results:**

Primary care centres with care as usual had a lower proportion of sick listed patients with CMD at both year 1 and 2. Primary care centres with a care manager organisation (CMO) had significantly fewer mean number of sick leave days (net and gross days) among patients with CMD compared to centres with care as usual, indicating a lower impact of sick leave. Sick leave duration among sick listed patients did not show statistically significant differences between the two groups of primary care centres. Both groups of primary care centres increased their sick leave duration significantly from year 1 to year 2, congruent to Sweden as a whole.

**Conclusion:**

The aim of this study was to evaluate two measures of sick leave in primary care centres with a care manager organisation compared to care as usual. There were no differences in sick leave duration. Primary care centres with a care manager organisation, designed to increase accessibility and continuity for patients with CMD, seemed to facilitate the primary care centre’s possibility to offer enhanced care taking to more patients with CMD with continued lower levels of impact of sick leave compared to care as usual.

**Implementation:**

This study evaluated outcomes after implementation of CMO at primary care centres.

## Background

Common mental disorders (CMD - depression, anxiety syndromes and stress-related mental disorders) stand for an increasing proportion of the disease burden in Europe and the North Americas [1,2], with almost 18% of the working population affected annually [3]. In EU only, CMD costs are estimated to €600 billion each year [2], including direct costs for health care and sickness benefits, and indirect costs due to productivity and income losses [4,5].

In Sweden, the primary care and its primary care centres is the first line of mental health service with main responsibility for mild to moderate symptoms of CMDs [6], leaving more severe cases to specialised psychiatry care [7].

The care model with strongest evidence for identification and treatment of CMD, especially depressive and anxiety disorders, is the collaborative care model with a care manager (a specially trained care provider, most often one of the primary care centre’s nurses who works 20–30% as care manager) [8–11]. These models usually include an interdisciplinary approach with the care manager having the main responsibility for structured care management and regular follow-ups with patients (face-to-face, digitally or by telephone) and provide team-based care in a stepped care model plus further contacts with the general practitioner (GP) when needed [12,13]. Follow-up times varied between 6 weeks and 57 months and the most common follow-up times varied between 1 and 6 months [12]. In the more recent review [13] most trials had shorter follow-up times (max. 6 months) and longer follow-ups was suggested.

CMDs are the leading diagnostic group for sick leave among women and men in Sweden [14]. The effects of different interventions aiming to improve the care of CMDs, reduce sick leave frequency, sick leave duration and increase return-to-work (RTW) for this patient group have shown inconsistent results [15–17]. A meta-analysis concluded that psychological interventions such as Cognitive Behavioural Therapy (CBT), problem-solving therapy and collaborative care had positive effects on sick leave compared to usual care. Effect sizes were small and none of the interventions were superior to the others [18].

In the fall of 2015, the Region Västra Götaland (VG region) in South-West Sweden started to implement a care manager organisation in the regional primary care with the main objective to enhance access to, and continuity of, evidence-based care for patients with CMD [19]. Prior the implementation phase, a first randomised controlled trial (RCT) was conducted in the region in 2014 at a limited number of primary care centres [20]. The results of that RCT showed positive effects of the care manager organisation on symptom remission and earlier RTW [20]. Since 2017, it is mandatory for all primary care centres in the region to have a care manager organisation on site, and during 2015–2017 approximately 87% of the region’s primary care centres implemented a care manager organisation.

In the present study, we aimed to estimate the association between the care manager organisation and the impact of sick leave and sick leave duration in CMD diagnoses by the use of national and regional register data.

Our research questions were:

(1) assess the impact of sick leave in CMD diagnoses in primary care centres with and without a care manager organisation year 1 and year 2 after implementation, (2) assess changes in impact of sick leave at primary care centres from year 1 to year 2, (3) assess the sick leave duration in CMD diagnoses in primary care centres with and without a care manager organisation year 1 and year 2 and (4) assess changes in sick leave duration at primary care centres from year 1 to year 2.

## Methods

### Study design and setting

This study had an ecological study design with groups as the analytical level [21]. We used aggregated register data at primary care centre level. The study was conducted in the primary care in the VG region. The primary care in Sweden is the first line of care and stands for almost 17% of the total healthcare costs. The Swedish health care system is mainly funded by income-based taxes and offers universal coverage for the population. The state establishes the health care system’s rules and regulations but the 21 regions in Sweden are autonomous political entities responsible for financing, organising and delivering health care services to residents in its geographical area. This public responsibility is combined with a universal health coverage which is a strength when performing this kind of study. The VG region has a population of approximately 1.7 million people corresponding to 17% of the Swedish population. Nationally, there are around 1200 primary care centres and about 200 of these are located in the VG region (52% with publicly driven and 48% privately driven but contracted with and reimbursed by the VG region). The geographic catchment areas of the primary care centres play a role for the patient flow. In areas with few primary care centres, mainly rural areas, the demographic and socioeconomic distribution of patients does not vary between publicly or privately driven centres. In urban areas, the demographic and socioeconomic distribution of inhabitants is geographically unequal which also mirrors the patients at different centres. In the VG region there is only one major urban area centred around the second largest city in Sweden, Göteborg. The number of primary care centres are higher in more affluent areas and these are mainly privately driven.

### The care manager organisation

The implementation process of the care manager organisation starts with a web-based workshop and education for the primary care centre’s staff including information about the national treatment guidelines for CMD. The next step includes an education of a nurse, employed at the primary care centre in CMD management. The care manager nurse works in close collaboration with the treating GP and other professions such as psychologists, psychotherapists, counsellors, physiotherapists[20].

### Usual care

Usual care for CMD can consist of several different components most often including psychological treatments (mainly Cognitive Behavioural Therapy), antidepressants and/or sick listing [7]. In the present study usual care means the care given at a primary care centre without a care manager organisation.

### Participating primary care centres and study periods

All primary care centres in the VG region during the years 2015 to 2017 were included. The study periods were 1 September 2015 to 31 August 2016 (the first year of implementation, hereafter called year 1) and 1 September 2016 to 31 August 2017 (the second year after implementation, hereafter called year 2).

### Register data

The patient group of interest, measured per primary care centre, included women and men 18–66 years with ICD-10 diagnoses: depression (F32, F33), anxiety syndromes (F40, F41) and stress-related mental disorders (F43 excluding post-traumatic stress disorder).

Aggregated data on sick leave in the CMD patient group at each primary care centre was obtained from the database MIDAS (Micro Data for Analysis of the Social Insurance) managed by the Swedish Social Insurance Agency. The data included information at primary care centre level (not per individual patient). Additional data on primary care centre characteristics (number of listed patients, type of management and rural or urban establishment) was collected from the regional health care database VEGA managed by the Department of Data and Analysis at the VG region. The data from both registers were merged to facilitate analyses.

### Outcome measures

Two ecological measures of sick leave with CMD were chosen as outcome variables. The first measure was primary care centre’s impact of sick leave (irrespective of certification diagnosis) in the total patient population (all patients with a CMD diagnosis at the primary care centre), stratified by sex (female and male patient population).

Impact of sick leave was calculated with the following equation:

$$\frac{\text{Mean}\;no.\;\text{of}\;\left(\text{net},\text{gross}\right)\;\text{sick}\;\text{leave}\;\text{days}\;\text{in}\;\text{sick}\;\text{listed}\;\text{patients}\;\text{with}\;\text{CMD}\;\text{per}\;\text{primary}\;\text{care}\;\text{centre}}{\text{Mean}\;\text{no}.\;\text{of}\;\text{patients}\;\text{with}\;\text{CMD}\;\text{per}\;\text{primary}\;\text{care}\;\text{centre}\;}$$

=Primary  care  centre′s  impact  of  sick  leave  in  patient  population  with  CMD

Sick leave duration was operationalised in two ways to facilitate a kind of aggregated sensitivity analysis: (1) mean number of net days of sick leave (number of sick leave days converted into whole days, e.g. 2 days on 50% sick leave correspond to one net sick leave day) and (2) mean number of gross days of sick leave (number of sick leave days regardless of extent of sick leave). Sick leave duration was calculated per primary care centre (stratified by sex) and based on patients with a CMD diagnosis and sick listed irrespective of certification diagnosis. Sick leave duration was calculated with the following equation:

$$\frac{\text{Mean}\;\text{no}.\;\text{of}\;\left(\text{net},\;\text{gross}\right)\;\text{sick}\;\text{leave}\;\text{days}\;\text{in}\;\text{sick}\;\text{listed patients}\;\text{with}\;\text{CMD}\;\text{per}\;\text{primary}\;\text{care}\;\text{centre}}{\text{Total}\;\text{no}.\;\text{of}\;\text{sick}\;\text{listed}\;\text{patients}\;\text{with}\;\text{CMD}\;\text{per}\;\text{primary care}\;\text{centre}\;}$$

=Sick  leave  duration  in  sick  listed  patients  with  CMD  per  primary  care  centre

### Analytical sample and covariates

The primary care centres were divided into two groups based on whether they had implemented a care manager organisation or not identified through the registration of a specific care manager code: (1) Primary care centres with a care manager organisation during the complete study period, and (2) Primary care centres that had not implemented the organisation within the study period and thus offered the patients usual care services. The following variables were included as covariates because of potential confounding effect with the outcome measures: 1. Type of management (public or private), 2. Care Need Index (CNI, www.scb.se), an index based on data on the primary care centres’ listed patients which measures the socioeconomic burden according to set levels with additional weighted reimbursements from the region (grouped into none/low (<20,000 SEK), medium (20,000–100,000 SEK) or high (>100,000 SEK)) and 3. Proportion of sick listed patients with CMD of total patient group with CMD.

### Statistical analysis

Mann-Whitney *U*-test was applied to descriptively assess group differences in the two non-parametric independent samples (with or without care manager organisation). The descriptive statistics were presented in proportions and median values with corresponding *p*-values due to a skewed and non-normal distribution. A linear mixed-effects model was implemented to investigate cross-sectional group differences and longitudinal changes in impact of sick leave and sick leave duration. The model assesses potential correlations between and within the two groups of primary care centres by applying repeated measures of fixed effects [22]. The analysis included care manager status (reference = primary care centres with usual care), study period (reference = year 1), the interaction between care manager status and study period, and with the sick leave measures as dependent variables. The analysis was further adjusted for Type of management (Model I), + CNI (Model II), + Proportion of sick listed patients with CMD of total CMD patient group at the primary care centre (Model III). The results of the linear mixed-effects model are presented in mean values and mean differences with 95% confidence intervals for between and within group differences. A generalised linear regression model with Type III sums of squares was applied to analyse potential interaction effects between care manager status and the outcome measures with the descriptive variables Type of management and Number of listed patients.

The analyses were conducted with IBM SPSS Statistics^®^ version 27 and SAS software version 9.4 (SAS Institute, Cary, NC.). Statistical significance was 0.05 (2-sided tests) with 95% confidence interval by default [22].

## Results

The categorisation of primary care centres into usual care and care manager organisation led to the exclusion of 31 primary care centres that either had implemented a care manager organisation during the follow-up year (*n* = 24) or that had a care manager only during the first year and not the second year (*n* = 7). The final sample (*n* = 172) included 91 primary care centres with usual care s and 81 primary care centres with a care manager organisation during the complete study period of two years.

The descriptive statistics showed that primary care centres with a care manager organisation to a higher extent were publicly driven (65% vs 43%, *p* < 0.01), and had more listed patients (*p* < 0.05 for year 1 and *p* < 0.01 for year 2) compared to primary care centres without a care manager organisation (Table 1). There were differences in the proportion of sick listed female patients per primary care centre with a lower level at the primary care centres with care as usual (*p* < 0.05 for year 1 and *p* < 0.01 for year 2). The differences were non-significant in both years for the male patients. An additional analysis of longitudinal change showed that both groups of primary care centres decreased their proportion of sick listed patients with CMD in year 2 compared to year 1 (*p* < 0.001 for men and women separately, data not shown).

**Table 1.** Description of the two different primary care Centre groups (with and without care manager organisation) year 1^a^ and year 2^b^: Management, location, number of listed patients, proportion of patients with common mental disorders, diagnosis, care manager contact and proportion of patients sick listed with diagnosis common mental disorder.

	Year 1			Year 2		
	Non-CMO^+^	CMO^+^^+^		Non-CMO	CMO	
	n (%)	n (%)	*p*	n (%)	n (%)	*p*
Type of management						
Public	39 (43)	53 (65)	0.004**	39 (43)	53 (65)	0.004**
Private	52 (57)	28 (35)		52 (57)	28 (35)	
Geographical location^1^						
Urban	76 (84)	69 (85)	0.84	77 (85)	69 (85)	1.00
Rural	15 (16)	12 (15)		14 (15)	12 (15)	
PCCs with no. of listed patients			0.02*			0.009**
<5000	21 (26)	11 (14)		26 (31)	11 (14)	
5000 - <7000	16 (19)	9 (11)		13 (16)	9 (11)	
7000 - <10.000	23 (28)	30 (38)		23 (27)	32 (40)	
≥ 10.000	22 (27)	30 (38)		22 (26)	29 (36)	
Median (min-max)	8061 (252–16,159)	9020 (2390–1894)	0.02*	8078 (503–15,704)	8932 (2667–18,504)	0.01**
Mean proportion of patients with CMD, per PCC^2^:	% (SE)	% (SE)		% (SE)	% (SE)	
Women	14.1 (1.1)	12.8 (1.1)	0.40	13.5 (0.7)	12.8 (0.7)	0.47
Men	7.2 (0.7)	6.2 (0.8)	0.36	6.8 (0.5)	6.2 (0.5)	0.38
Mean proportion of patients with CMD who had ≥1 visit(s) to a care manager, per PCC^3^:	% (SD)	% (SD)		% (SD)	% (SD)	
Women	0 (0)	1.5 (2.0)	<0.001***	0 (0)	3.2 (3.3)	<0.001***
Men	0 (0)	1.2 (1.5)	<0.001***	0 (0)	2.9 (2.9)	<0.001***
Mean proportion of sick listed patients with CMD, per PCC^4^:	% (SE)	% (SE)		% (SE)	% (SE)	
Women	19.4 (0.5)	21.0 (0.5)	0.04*	15.3 (0.5)	17.2 (0.5)	0.006**
Men	16.0 (0.5)	17.2 (0.5)	0.07	11.9 (0.4)	13.0 (3.3)	0.06

^a^September 2015–August 2016.

^b^September 2016–August 2017.

^+^Non-CMO, without care manager organisation.

^++^CMO, care manager organisation.

^1^Measured by whether the PCCs had received earmarked reimbursement for their geographical location (e.g. distant location from large city or hospital, location on an island without bridge connection, etc.) during the study period.

^2^Mean proportion = mean number of patients with CMD divided by mean number of listed patients at each PCC.

^3^Mean proportion = mean number of patients with CMD who had at least one visit to a care manager divided by mean number of patients with CMD at each PCC.

^4^Mean proportion = mean number of sick listed patients with CMD divided by mean number of patients with CMD at each PCC.

*p-value <0.05, **p-value <0.01, ***p-value <0.001.

### Impact of sick leave in the two groups of primary care centres

The cross-sectional analysis showed that primary care centres with a care manager organisation had significantly lower impact of sick leave (*p* < 0.05 for net days and *p* < 0.01 for gross days in female sick leave and *p* < 0.05 for gross days year 1, otherwise *p* < 0.01 in male sick leave) compared to primary care centres offering usual care (Table 2). Primary care centres with a care manager organisation showed stronger statistically significant increase in impact of sick leave over time compared to primary care centres with usual care. Adjusting for type of management (model I) did not alter the results but the adjustments for type of management and CNI (model II) decreased the differences between the two groups of primary care centres in all outcomes (Appendix 1). The group differences were still statistically significant at the 0.05 level for all outcomes except for impact of sick leave operationalised as net sick leave days in female sick leave year 1 (*p* = 0.066) and operationalised as gross sick leave days in male sick leave year 1 (*p* = 0.055). Adding proportion of sick listed patients of total patient population to the model adjustment (model III) further decreased the differences between the two groups as well as the differences within each group of primary care centres from year 1 to year 2 but did not eliminate any statistically significant associations.

**Table 2.** Impact of sick leave^a^ among all patients with common mental disorders by primary care centre with and without care manager organisation, year 1^b^ and year 2^c^.

	Impact of sick leave (Net^d^ sick leave days)			Impact of sick leave (Gross^e^ sick leave days)		
	Non-CMO ^+^	CMO^+^^+^		Non-CMO	CMO	
Crude, *n* = 167	Mean	Mean	Diff. (CI^^^)	Mean	Mean	Diff. (CI^^^)
*Women*						
Year 1	29.1	18.5	−10.7 (-19.1; −2.2)*	35.1	22.4	−12.7 (-21.8; −3.7)**
Year 2	32.0	24.7	−7.3 (-12.9; −1.6)*	39.6	30.9	−8.7 (-15.1; −2.2)**
Diff. (CI^^^)	2.8 (-0.7; 6.3)	6.2 (2.6; 9.8)**		4.5 (0.7; 8.3)*	8.5 (4.6; 12.4)***	
*Men*						
Year 1	57.1	39.2	−17.9 (-31.3; −4.5)**	65.9	45.8	−20.1 (-35.7; −4.5)*
Year 2	67.5	52.2	−15.4 (-25.5; −5.3)**	80.5	63.0	−17.5 (-29.6; −5.4)**
Diff. (CI^^^)	10.5 (2.4; 18.6)*	13.0 (4.8; 21.3)**		14.6 (5.3; 23.4)**	17.1 (7.7; 26.5)**	

^a^Impact of sick leave (irrespective of certification diagnosis) was calculated as net and gross sick leave days per patient in the total patient population (all patients with a CMD diagnosis at the primary care centre and not only those sick listed).

^b^September 2015 – August 2016.

^c^September 2016 – August 2017.

^d^Net sick leave days = number of sick leave days converted into whole days, e.g. 2 days on 50% sick leave correspond to one net sick leave day.

^e^Gross sick leave days = number of sick leave days regardless of extent of sick leave.

^+^Non-CMO, without care manager organisation.

^++^CMO, care manager organisation.

^^^CI, 95% confidence interval.

*p-value <0.05, **p-value <0.01, ***p-value <0.001.

### Sick leave duration per sick listed patients with CMD and group of primary care centres

We found no statistically significant differences in sick leave duration during the study period between the primary care centres with care as usual compared to those with care manager organisation (Table 3). The longitudinal analysis showed strong statistically significant increases in mean days of sick leave (both net and gross days) in female and male sick leave in the two groups from year 1 to year 2 (*p* < 0.0001 for all longitudinal outcomes). Male sick leave had somewhat higher increase in mean days compared to female sick leave in the two sick leave measures in both groups of primary care centres. The mean values changed only slightly when adjusting for type of management (model I), CNI (model II) or proportion of sick listed patients with CMD of total patient population (model III) (see Appendix 2). The adjustments did not change the statistical significance in any of the outcomes.

**Table 3.** Sick leave duration^a^ per sick listed patient with common mental disorders by primary care centre group, year 1^b^ and year 2^c^, with and without care manager organisation.

	Sick leave duration (Net^d^ sick leave days)			Sick leave duration (Gross^e^ sick leave days)		
	Non-CMO^+^	CMO^+^^+^		Non-CMO	CMO	
Crude, *n* = 169	Mean	Mean	Diff. (CI^^^)	Mean	Mean	Diff. (CI^^^)
Women						
Year 1	78.7	81.2	2.5 (-1.5; 6.5)	96.0	98.7	2.7 (-1.7; 7.1)
Year 2	111.4	114.9	3.6 (-1.8; 8.9)	139.8	144.5	4.7 (-1.3; 10.7)
Diff. (CI^^^)	32.7 (29.5; 35.9)***	33.7 (30.4; 37.1)***		43.8 (40.0; 47.6)***	45.8 (41.9; 49.7)***	
Men						
Year 1	81.4	81.9	0.5 (-5.3; 6.2)	93.9	95.9	2.0 (-4.2; 8.2)
Year 2	119.6	120.6	1.1 (-7.0; 9.1)	142.0	144.9	2.9 (-6.3; 12.1)
Diff. (CI^^^)	38.1 (32.9; 43.3)***	38.7 (33.3; 44.1)***		48.1 (42.0; 54.2)***	49.0 (42.6; 55.3)***	

^a^Sick leave duration was calculated as net and gross sick leave days per sick listed patient with CMD and irrespective of sick leave diagnoses.

^b^September 2015–August 2016.

^c^September 2016–August 2017.

^d^Net sick leave days = number of sick leave days converted into whole days, e.g. 2 days on 50% sick leave correspond to one net sick leave day.

^e^Gross sick leave days = number of sick leave days regardless of extent of sick leave.

^+^Non-CMO, without care manager organisation.

^++^CMO, care manager organisation.

^^^CI, 95% confidence interval.

***Statistically significant difference between years with a *p-*value of <0.001.

The association between the sick leave measures and care manager status did not differ between primary care centres with public or private management or among primary care centres with higher or lower numbers of listed patients (*p*-value of interaction effects were >0.05 for all outcomes, data not shown).

## Discussion

This study evaluated 2 years of a care manager organisation in regional primary care in South-West Sweden. Primary care centres with a care manager organisation had a lower impact of sick leave, as their patient population (all patients with CMD regardless of sick leave status) had fewer mean numbers of sick leave days, compared to primary care centres offering usual care. This was apparent in female and male sick leave and in net and gross sick leave days.

Earlier evaluations of the care manager organisation in the VG region found a more efficient care taking through sustained or improved treatment regarding antidepressant use [23] and length of psychotherapy treatment [24]. Enhanced care taking could explain the primary care centres’ lower impact of sick leave found in the present study. A study [20] conducted on the individual level with fewer primary care centres included, found that care managers compared to usual care improved symptom remission of depression and reduced time to partial RTW indicating an enhanced care taking with a care manager organisation on site.

No statistically significant differences were found between the two groups of primary care centres in sick leave duration. The measure relates mean number of sick leave days specifically to sick listed individuals with CMD. A lack of effect on sick leave duration was reported in a recent Cochrane review concluding that interventions in primary care only found moderate or no evidence of effects on RTW [17].

The lack of differences in sick leave duration could probably be explained by the GP:s being adherent to the recommendations of sick leave duration in the Insurance Medical Decision Support, regulated by the Swedish National Board of Health and Welfare [25]. In this study the care manager organisation was evaluated at an aggregated level with register data. It was not possible to analyse how well the need of sick listing was met in the individual patient. In comparison with national statistics the sick leave duration matches the overall sick leave duration with CMD for the whole country [14].

Another possible explanation is the introduction of a collaboration project in the VG region during the study period. The project implemented a new working model and new care function (rehabilitation coordinator) for a more quality-assured and efficient rehabilitation and sick leave process in primary care [26]. The presence of rehabilitation coordinator at the primary care centres could have led to a more homogenous sick listing procedure.

Another finding was that primary care centres with and without a care manager organisation increased the mean number of sick leave days from year 1 to year 2. The increase is congruent to Swedish national data [14], also mirroring European data.

### Methodological considerations

The present register-based study was designed in accordance with the STROBE guidelines [27]. The use of high-quality register data from the Swedish social insurance implies low risk of bias such as high attrition levels, incomplete data, selection bias or information fallacies, more common in studies with self-reported data [28]. Another strength is that the data emerge from clinical practice and the results can be re-applied in the everyday clinical setting, attenuating the gap between research and practice. The main limitation is that data is collected by an external part without possibilities for researchers to control for confounding variables or assess data quality [28]. A limitation with aggregated register data is that we lack information from the individual such as symptom severity or work situation. This is of importance in CMD diagnoses as mild symptoms in most cases do not require sick listing. To counteract this factor, we chose to include two types of sick leave measures, one with the total patient population with CMD and one only including those that were sick listed.

Furthermore, external factors, such as sociodemographic and socioeconomic status, and workplace interaction are important in the RTW-process. With this study design these individual factors were not possible to control for. We did control for CNI which is based on the primary care centres’ socioeconomic patient data and did not find that this factor influenced the results in any way or was unevenly distributed in the two groups of primary care centres.

One limitation with the use of MiDAS is that it only records sick leave periods exceeding 14 days (among employed persons), shorter periods are reimbursed by the employer and not eligible for sick leave benefits from the Swedish Social Insurance Agency. This could result in a rather crude measurement of sick leave duration if many sick leave periods are 13 days or shorter. However, patients with CMD are usually sick listed for longer periods of time compared to other patient groups [25]. Recent statistics from the Swedish Social Insurance Agency state that sick leave spells in CMD diagnoses are in median 90 days long, for both men and women [14]. Hence, we do not believe that this factor influenced our results in any significant way.

Notable is also the change in the Swedish sickness insurance regulation in 2016 where a former time limit for duration of a sick leave spell was abolished. This was combined with an action program from the Government to the Swedish Social Insurance Agency. The program included several different types of actions with a main goal to avoid an increase of sick leave durations as an effect of the abolished time limit and to reach a set goal of 9.0 sickness benefit days at the national level. The Swedish Social Insurance Inspectorate published an evaluation of consequences of changes in sickness insurance regulations in 2023 [29]. The report concluded that an increase in sickness benefit days after abolishing the time limit in 2016 did not occur at the expected level [29]. However, it is important to bear in mind that changes in regulations may affect research studies. In this case, any effect of changes would have affected the primary care centres in similar ways and irrespective of care management organisation or care as usual.

### Implications

Mental health problems have increased largely as main reason for reduced work capacity in many European countries [30] and now constitute large societal and individual costs, including increased expenditures on welfare systems and loss of income [31]. A care manager organisation is a complex intervention, and it is of great importance to follow and evaluate development of new organisational health care reforms as a form of quality assurance. These evaluations are made possible by compiling and analysing register data on regional as well as national level.

A collaborative care model with a care manager has been evaluated on an individual level in several international studies [8,13,32–34], and our belief is that the findings from the present study on a Swedish care manager organisation could add to the knowledge base concerning effective care taking of patients with CMD in primary care.

## Conclusions

The aim of this study was to evaluate two measures of sick leave in primary care centres with or without a care manager organisation. There were no differences in sick leave duration. Primary care centres with a care manager organisation, designed to increase accessibility and continuity for patients with CMD, seems to facilitate the primary care centre’s possibility to offer enhanced care taking to more patients with CMD with continued lower levels of impact of sick leave compared to usual care.

## Supplementary Material

Appendix jan.docx

## Data Availability

The datasets generated and/or analysed during the current study are not publicly available due to Swedish legislation but are available from the corresponding author on reasonable request.
