# Supplementary material for: Care manager organisation in Swedish primary care centres: impact of sick leave and sick leave duration in patients with common mental disorders. A register-based study
Source: Scand J Prim Health Care. 2025 Mar 14;43(3):576–84. doi: 10.1080/02813432.2025.2477150 (PMC12377119; doi:10.1080/02813432.2025.2477150)
Supplement: Appendix jan.docx [file IPRI_A_2477150_SM2177.docx]

| Appendix 1. Adjusted impact of sick leave^a^ among all patients with common mental disorders by primary care center with and without care manager organisation, year 1^b^ and year 2^c^ | | | | | | | |
| --- | --- | --- | --- | --- | --- | --- | --- |
|  | **Net^d^ sick leave days** | | | **Gross^e^ sick leave days** | | | |
|  | Non-CMO^+^ | CMO^++^ |  | Non-CMO | CMO |  | |
| **Model I, n=167** | Mean | Mean | Diff. (CI^^^) | Mean | Mean | Diff. (CI^^^) | |
| *Women* |  |  |  |  |  |  | |
| Year 1 | 29.2 | 18.4 | -10.7 (-19.2; -2.2)^*^ | 35.2 | 22.3 | -12.9 (-22.1; -3.7)^**^ | |
| Year 2 | 32.0 | 24.6 | -7.4 (-13.2; -1.5)^*^ | 39.7 | 30.8 | -8.9 (-15.5; -2.3)^**^ | |
| Diff. (CI^^^) | 2.8 (-0.7; 6.3) | 6.2 (2.6; 9.8)^**^ |  | 4.5 (0.7; 8.3)^*^ | 8.5 (4.6; 12.4)^***^ |  | |
| *Men* |  |  |  |  |  |  | |
| Year 1 | 56.9 | 39.3 | -17.6 (-31.2; -4.1)^*^ | 65.9 | 45.9 | -20.0 (-35.8; -4.2)^*^ | |
| Year 2 | 67.4 | 52.3 | -15.1 (-25.5; -4.7)^**^ | 80.4 | 63.0 | -17.4 (-29.8; -4.9)^**^ | |
| Diff. (CI^^^) | 10.5 (2.4; 18.6)^*^ | 13.0 (4.8; 21.3)^**^ |  | 14.5 (5.3; 23.7)^**^ | 17.1 (7.7; 26.5)^**^ |  | |
| **Model II, n=146** | |  |  |  |  |  | |
| *Women* |  |  |  |  |  |  | |
| Year 1 | 27.5 | 18.8 | -8.7 (-18.0; 0.6) | 32.5 | 22.7 | -9.8 (-19.5; -0.2)^*^ | |
| Year 2 | 31.7 | 24.9 | -6.7 (-13.2; -0.3)^*^ | 38.8 | 31.2 | -7.6 (-14.8; -0.4)^*^ | |
| Diff. (CI^^^) | 4.2 (0.3; 8.0) ^*^ | 6.1 (2.3; 9.9)^**^ |  | 6.3 (2.3; 10.3)^**^ | 8.5 (4.6; 12.5)^***^ |  | |
| *Men* |  |  |  |  |  |  | |
| Year 1 | 54.6 | 39.4 | -15.1 (-29.9; -0.4)^*^ | 63.2 | 46.2 | -17.0 (-34.4; 0.4) | |
| Year 2 | 65.5 | 53.0 | -12.6 (-23.6; -1.6)^*^ | 78.5 | 64.0 | -14.5 (-27.7; -1.2)^*^ | |
| Diff. (CI^^^) | 11.0 (1.7; 20.2)^*^ | 13.5 (4.4; 22.6)^**^ |  | 15.3 (4.7; 25.9)^**^ | 17.9 (7.5; 28.3)^**^ |  | |
| **Model III, n=146** | |  |  |  |  |  | |
| *Women* |  |  |  |  |  |  | |
| Year 1 | 28.0 | 19.6 | -8.4 (-17.6; 0.8) | 33.3 | 24.0 | -9.3 (-18.8; 0.2) | |
| Year 2 | 30.9 | 24.5 | -6.3 (-12.7; 0.05) | 37.4 | 30.5 | -6.9 (-14.0; 0.1) | |
| Diff. (CI^^^) | 2.9 (-1.7; 7.4) | 4.9 (0.5; 9.4)^*^ |  | 4.2 (-0.7; 9.0) | 6.5 (1.8; 11.2)^**^ |  | |
| *Men* |  |  |  |  |  |  | |
| Year 1 | 53.8 | 38.4 | -15.4 (-30.2; -0.6)^*^ | 62.3 | 45.0 | -17.4 (-34.8; 0.1) | |
| Year 2 | 66.5 | 53.7 | -12.8 (-23.8; -1.7)^*^ | 79.6 | 64.9 | -14.7 (-28.0; -1.4)^*^ | |
| Diff. (CI^^^) | 12.8 (2.3; 23.2)^*^ | 15.4 (4.9; 25.8)^**^ |  | 17.3 (5.3; 29.3)^**^ | 20.0 (7.9; 32.0)^**^ |  | |
| ^a^Impact of sick leave (irrespective of certification diagnosis) was calculated as net and gross sick leave days per patient in the total patient population (all patients with a CMD diagnosis at the primary care centre and not only those sick listed).  ^b^September 2015 – August 2016, ^c^September 2016 – August 2017  ^d^Net sick leave days = number of sick leave days converted into whole days, e.g. two days on 50% sick leave correspond to one net sick leave day  ^e^Gross sick leave days = number of sick leave days regardless of extent of sick leave  ^+^Non-CMO, without care manager organisation, ^++^CMO, care manager organisation, ^^^CI, 95% confidence interval  ^*^*P-*value <0.05, ^**^*P-*value <0.01, ^***^*P-*value <0.001  Model I (adjusted for type of management)  Model II (adjusted for Model I + Care Need Index ‘CNI’)  Model III (adjusted Model II + proportion of patients with sickness benefits of total study population) | | | | | | |  |

| Appendix 2. Adjusted sick leave duration^a^ per sick listed patient with common mental disorders by primary care center group, year 1^b^ and year 2^c^, with and without care manager organisation | | | | | | |
| --- | --- | --- | --- | --- | --- | --- |
|  | **Net^d^ sick leave days** | | | **Gross^e^ sick leave days** | | |
|  | Non-CMO^+^ | CMO^++^ |  | Non-CMO | CMO |  |
| **Model I, n=169** | Mean | Mean | Diff. (CI^^^) | Mean | Mean | Diff. (CI^^^) |
| *Women* |  |  |  |  |  |  |
| Year 1 | 78.9 | 81.0 | 2.1 (-2.0; 6.2) | 96.3 | 98.3 | 2.1 (-2.4; 6.5) |
| Year 2 | 111.6 | 114.7 | 3.2 (-2.2; 8.6) | 140.1 | 144.1 | 3.0 (-2.0; 10.1) |
| Diff. (CI^^^) | 32.7(29.5; 35.9)^***^ | 33.7(30.4; 37.1)^***^ |  | 43.8(40.0; 47.6)^***^ | 45.8(41.9; 49.7)^***^ |  |
| *Men* |  |  |  |  |  |  |
| Year 1 | 82.0 | 81.3 | -0.6 (-6.4; 5.2) | 94.4 | 95.4 | 0.9 (-5.3; 7.3) |
| Year 2 | 120.1 | 120.0 | -0.1 (-8.2; 8.1) | 142.5 | 144.4 | 1.8 (-7.2; 11.1) |
| Diff. (CI^^^) | 38.1 (32.9;43.3)^***^ | 38.7 (33.3; 44.1)^***^ |  | 48.1 (42.0; 54.2)^***^ | 49.0 (42.6; 55.3)^***^ |  |
| **Model II, n=146** | |  |  |  |  |  |
| *Women* |  |  |  |  |  |  |
| Year 1 | 78.7 | 81.2 | 2.5 (-2.0; 7.1) | 95.6 | 98.5 | 2.8 (-1.9; 7.6) |
| Year 2 | 111.9 | 114.4 | 2.5 (-3.2; 8.3) | 140.4 | 143.8 | 3.4 (-2.8; 9.7) |
| Diff. (CI^^^) | 33.2 (29.6;36.7)^***^ | 33.2 (29.7; 36.7)^***^ |  | 44.7 (40.5;48.9)^***^ | 45.3 (41.3;49.4)^***^ |  |
| *Men* |  |  |  |  |  |  |
| Year 1 | 82.2 | 80.2 | -2.0 (-8.8; 4.7) | 94.4 | 94.3 | -0.05 (-7.5; 7.5) |
| Year 2 | 119.2 | 119.5 | 0.3 (-7.6; 8.1) | 142.3 | 144.2 | 1.8 (-6.9; 10.6) |
| Diff. (CI^^^) | 37.0 (31.5;42.6)^***^ | 39.4 (33.9; 44.8)^***^ |  | 48.0 (41.4; 54.5)^***^ | 49.8 (43.4; 56.3)^***^ |  |
| **Model III, n=146** | |  |  |  |  |  |
| *Women* |  |  |  |  |  |  |
| Year 1 | 78.6 | 81.1 | 2.5 (-2.1; 7.1) | 95.6 | 98.4 | 2.8 (-1.9; 7.6) |
| Year 2 | 112.0 | 114.5 | 2.5 (-3.3; 8.2) | 140.4 | 143.8 | 3.4 (-2.9; 9.8) |
| Diff. (CI^^^) | 33.4 (29.4;37.4)^***^ | 33.7 (30.4; 37.1)^***^ |  | 44.8 (40.1; 49.4)^***^ | 45.4 (40.9; 49.8)^***^ |  |
| *Men* |  |  |  |  |  |  |
| Year 1 | 80.3 | 77.5 | -2.8 (-9.4; 3.8) | 92.1 | 91.2 | -0.9 (-8.2; 6.4) |
| Year 2 | 121.8 | 121.5 | -0.4 (-8.1; 7.3) | 145.3 | 146.4 | 1.1 (-7.6; 9.7) |
| Diff. (CI^^^) | 41.6 (35.3;47.8)^***^ | 44.0 (37.8; 50.1)^***^ |  | 53.2 (45.9; 60.5)^***^ | 55.2 (48.0; 62.3)^***^ |  |
| ^a^Sick leave duration was calculated as net and gross sick leave days per sick listed patient with CMD and irrespective of sick leave diagnoses.  ^b^September 2015 – August 2016, ^c^September 2016 – August 2017  ^d^Net sick leave days = number of sick leave days converted into whole days, e.g. two days on 50% sick leave correspond to one net sick leave day  ^e^Gross sick leave days = number of sick leave days regardless of extent of sick leave  ^+^Non-CMO, without care manager organisation, ^++^CMO, care manager organisation, ^^^CI, 95% confidence interval  Model I (adjusted for type of management)  Model II (adjusted for Model I + Care Need Index ‘CNI’)  Model III (adjusted Model II + proportion of patients with sickness benefits of total study population)  ^***^Statistically significant difference between years with a *p-*value of <0.001. | | | | | | |
